# Use of sequential lateral flow assays to diagnose cryptococcal infection among people living with HIV in Monrovia, Liberia

**DOI:** 10.1371/journal.pntd.0013008

**Published:** 2025-04-08

**Authors:** Yassah M. Barclay-Korboi, Alina Adeel, Ibrahim Ajami, Flinhway Hessou Dickson, Ian Wachekwa, Nyenyakar A. F. Vaye, Stuart M. Levitz

**Affiliations:** 1 Department of Internal Medicine, John F. Kennedy Memorial Hospital, Monrovia, Liberia; 2 Faculty of Internal Medicine, A. M. Dogliotti College of Medicine, College of Health Sciences, University of Liberia, Monrovia, Liberia; 3 Department of Medicine, University of Massachusetts Chan Medical School, Worcester, Massachusetts, United States of America; Albert Einstein College of Medicine, UNITED STATES OF AMERICA

## Abstract

Cryptococcal meningitis is one of the top causes of morbidity and mortality in people living with HIV/AIDS. In high prevalence regions, current recommendations are to screen individuals with blood CD4^+^ T cell counts less than 200 cells/µl for serum cryptococcal antigen (CrAg) and then preemptively treat those who test positive for presumed cryptococcosis. However, in many low-resource settings, including Monrovia, Liberia, flow cytometric CD4 assays are not readily available. We tested subjects with known HIV infection using a lateral flow assay (LFA), which provides a semi-quantitative determination of whether the blood CD4^+^ T cell count is ≤200 cells/µl. Subjects with counts ≤200 cells/µl were then tested with an LFA that detects CrAg. Of the 500 HIV+ subjects tested, 201 (40.2%) had blood CD4^+^ T cell count ≤200. Of those, 82/201 (40.7%) were serum CrAg+. Subjects who were serum CrAg+ were more likely to have a Glasgow Coma Score <15, whereas subjects who were CrAg- were more likely to be HIV-2+. Lumbar punctures were performed on 61 serum CrAg+ subjects; 30/61 (49.2%) subjects were cerebrospinal fluid CrAg+. Thus, sequential point-of-care testing enabled the diagnosis of cryptococcosis in HIV+ individuals with blood CD4 T cell counts ≤200 cells/µl. As diagnostic testing informs life-saving therapies, it is imperative that these assays are made readily available in resource-poor settings.

## Author Summary

Brain infections due to the fungus Cryptococcus are estimated to kill over 100,000 persons infected with HIV annually. To detect disease early, it is recommended to screen the serum of individuals with low (≤200 cells/µl) blood CD4^+^ T cell counts for cryptococcal antigen (CrAg) and then preemptively treat those who test positive for presumed cryptococcosis. Unfortunately, flow cytometric CD4 assays are not readily available in many low-resource settings. To get around this limitation, we devised a strategy to test subjects with known HIV infection in Monrovia, Liberia, with a point-of-care lateral flow assay (LFA) which detects low blood CD4^+^ T cell counts. Those with low counts were then tested for serum CrAg using a second LFA. Of the 500 HIV+ Liberian study subjects tested, 201 had low blood CD4^+^ T cell counts. These subjects were further tested for serum CrAg; 82/201 tested CrAg+. Many of these subjects were then found to have cryptococcal meningitis. All subjects who were serum CrAg+ were treated with antifungal drugs. Thus, use of inexpensive, sequential point-of-care LFA testing enables early diagnosis and treatment of HIV-associated cryptococcosis. These lifesaving tests should be made widely available in resource-poor settings.

## Introduction

It is estimated that worldwide, 152,000 cases of HIV-associated cryptococcal meningitis occur annually, resulting in over 112,000 deaths [1]. The majority of the cases occur in Africa, often in resource-poor settings where access to diagnostic testing and the most effective antifungal drugs is limited [1, 2]. The encapsulated fungi *Cryptococcus neoformans* and its sister species *C. gattii* are the causative agents of virtually all cases of cryptococcosis.

During human infection, the polysaccharide capsule of *Cryptococcus*, known as cryptococcal antigen (CrAg), is shed. In persons infected with HIV, CrAg is usually detectable in blood prior to the onset of symptoms [3–5]. Current recommendations are to screen individuals with blood CD4^+^ T cell counts less than 100 cells/µl for CrAg and to consider screening those with CD4 count between 100-200 [5, 6]. Those who test positive should then be preemptively treated for presumed cryptococcosis. This guidance is supported by a randomized multicenter trial in Tanzania and Zambia which demonstrated a significant survival benefit to this preemptive treatment strategy [7]. However, in many resource-poor settings, quantitative testing of blood CD4^+^ T cell counts is not readily available due to the expense of flow cytometry reagents and the difficulty purchasing and maintaining flow cytometers.

Scant data exist on the prevalence of cryptococcosis in Liberia due to the scarcity of available testing [2,8]. The initial goal of the present study was to determine the prevalence of cryptococcal antigenemia in persons who are HIV+ with peripheral blood CD4^+^ T cell counts <200 cells/µl at John F. Kennedy Memorial Hospital (JFK Hospital), a large teaching and referral hospital in Monrovia, Liberia. However, shortly before the study was ready to begin, JFK Hospital lost its capacity to obtain CD4^+^ T cell determinations as their flow cytometer stopped working and could not be repaired. The study design was then changed; a lateral flow assay (LFA) that provides a semi-quantitative determination of whether the blood CD4^+^ T cell count is ≤200 cells/µl was purchased [9–11]. This LFA was used to screen HIV+ subjects; those with a screening test ≤200 CD4^+^ T cells/µl were then tested by an LFA that detects CrAg. With this relatively inexpensive sequential point of care testing algorithm, we were able to identify patients with advanced HIV disease who qualified for prophylaxis against opportunistic infections and preemptive treatment of cryptococcal disease.

## Methods

### Ethics statement

This prospective study was approved by the Institutional Review Board (IRB) of the University of Liberia, A.M. Dogliotti College of Medicine. Signed informed consent was obtained from all patients or their healthcare proxies. The client information sheet, consent form, study questionnaire and clinical data collection tool are included in the supplemental information (S1 Text).

### Study details

The study was conducted at the John F. Kennedy Medical Center, Liberia’s largest tertiary teaching hospital. Enrollment of subjects took place from October 2021 to December 2022. Subjects were either outpatients recruited from the facility’s Infectious Disease Clinic, with just over 3,000 active HIV+ clients, or inpatients from the Internal Medicine ward. HIV+ subjects aged >15 years were eligible to participate. Tests associated with the study and lumbar puncture kits were provided free of charge to participants. As testing for CD4 counts and CrAg was not otherwise available to treating physicians, a bias towards recruitment of sicker subjects, particularly those having signs or symptoms consistent with cryptococcosis, likely occurred. Consenting subjects were interviewed about their demographic traits and medical history. Clinical data and serostatus for HIV-1 and HIV-2 were obtained from chart reviews. A physical examination was performed, including measurement of the Glasgow Coma Score (GCS) [12].

Blood was tested by the Visitech CD4 Advanced Disease LFA (purchased from Omega Diagnostics Ltd., Alva, Scotland, UK). Subjects with ≤200 CD4 cells/µl as determined with the Visitech CD4 Advanced Disease LFA (CD4 LFA) were further tested for the presence of serum CrAg by the CrAg LFA (donated by IMMY, Norman, OK, USA). The diagnostic tests were used according to the manufacturers’ instructions. Representatives from Omega Diagnostics and IMMY provided technical training in the use of their products via live interactive webinars. Lumbar puncture was offered to patients who were CrAg+ as indicted based on WHO guidelines [5]. CrAg in the cerebrospinal fluid (CSF) was measured by CrAg LFA. As amphotericin B and 5-flucytosine were not generally available at JFK Hospital, fluconazole was prescribed to all CrAg+ subjects, in accordance with WHO guidelines [5]. Subjects with a positive CSF CrAg received fluconazole (for adults: induction therapy, 1200 mg/day for two weeks; consolidation therapy, 800 mg/d for eight weeks; and maintenance therapy, 200 mg/d).

CrAg- and CrAg+ subjects were compared using the two-sided Fisher’s exact test. Statistics were calculated and graphs were created using GraphPad Prism version 10.3.1.

## Results

### Characteristics of the study population based on serum cryptococcal antigenemia

**Fig 1:**
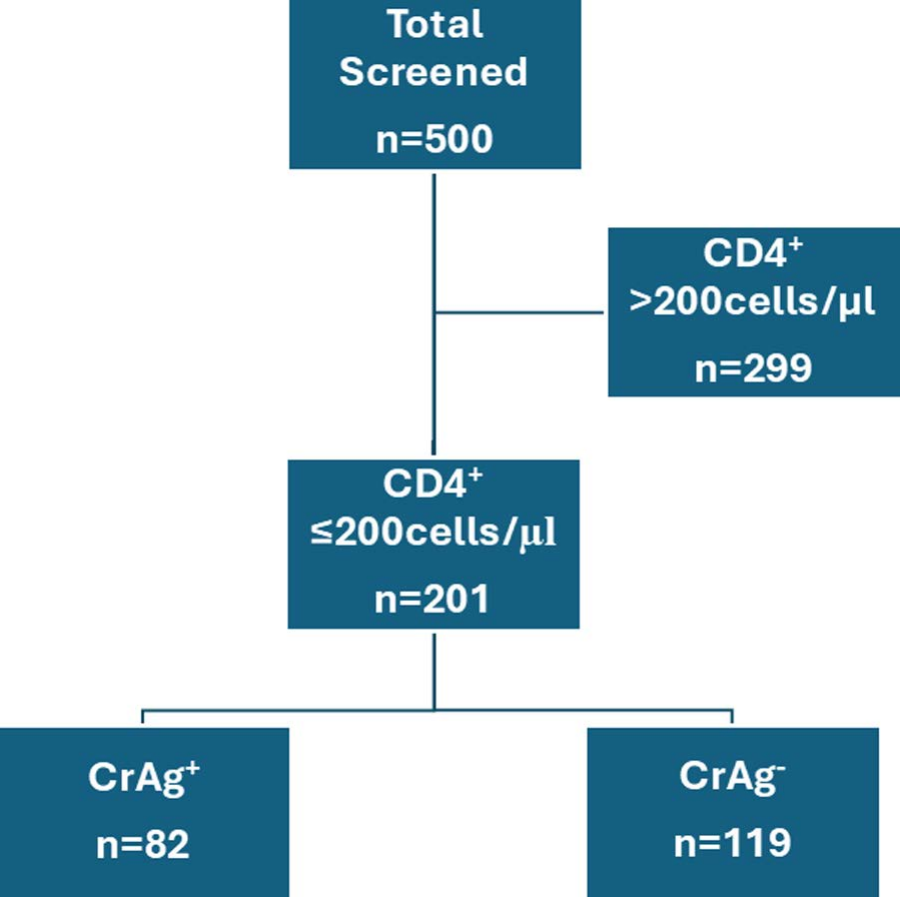
Study flow diagram. “n=” denotes the number of subjects in each group.

Of the 500 HIV+ individuals screened, 201 (40.2%) had ≤200 CD4 cells/µl using the Visitech CD4 Advanced Disease LFA (Fig 1). Of the 201 subjects, 86 were on antiretroviral therapy and 13 were receiving fluconazole upon study entry. Table 1 and S1 Table list selected demographic and laboratory characteristics of the 201 HIV+ subjects with CD4 counts ≤200 enrolled in the study. The serum CrAg test was positive in 82 (40.8%) subjects and negative in 119 (59.2%) subjects. The median age was 40 years old in both groups. There was a higher percentage of females than males in the study population; however, gender did not significantly differ as a function of CrAg status. HIV-1 and HIV-2 are endemic in West Africa [13]. Overall, 4/201 (2.0%) subjects were HIV-1-/HIV-2+ and 28/201 (13.9) subjects were dually positive with HIV-1 and HIV-2. HIV-2 seropositivity was inversely correlated with cryptococcal antigenemia. There was a trend, albeit not statistically significant, towards inpatients being more likely to be CrAg+. At day 30 following enrollment in the study, one (1.2%) and four (3.3%) CrAg+ and CrAg- subjects, respectively, had died. Due to a large number of subjects being lost to follow-up, we were unable to assess mortality beyond 30 days.

**Table 1:** Demographic and laboratory characteristics of subjects based on serum CrAg status.

		CrAg+	CrAg-	P value
Number of patients		82 (40.7%)	119 (59.2%)	
Median age (range)		40 (23 - 69 years)	40 (17 - 75 years)	
Gender	Male	22 (26.8%)	44 (36.9%)	0.17
	Female	60 (73.1%)	75 (63.0%)	
HIV-1+, HIV-2-		77 (93.9%)	92 (77.3%)	0.003
HIV-1-, HIV-2+		0 (0%)	4 (3.4%)	
HIV-1+, HIV-2+		5 (6.1%)	23 (19.3%)	
Inpatient		50 (61.0 %)	60 (50.4 %)	0.15

Abbreviations: CrAg+, positive for serum CrAg. CrAg-, negative for serum CrAg. P values calculated using Fisher’s exact test.

### GCS as a function of serum cryptococcal antigenemia

**Fig 2:**
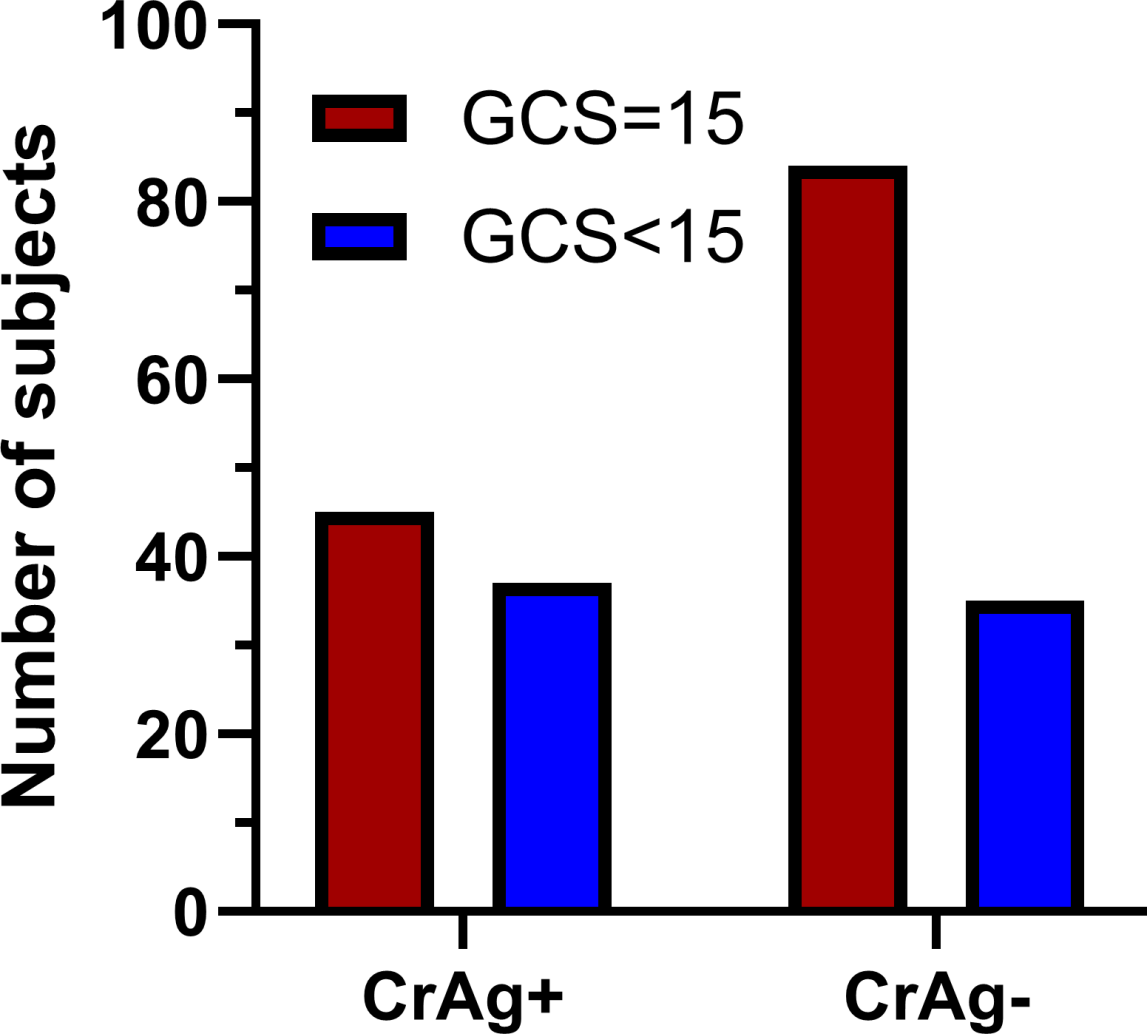
The relationship between level of consciousness and serum cryptococcal antigenemia. Subjects who were serum CrAg+ were more likely to have a GCS<15 (P=0.025 by the Fisher’s exact test).

The GCS was determined for all subjects upon study enrollment. Subjects were divided into those with a GCS of 15 (fully conscious) and those below 15 (semiconscious or comatose). Subjects who were CrAg+ were significantly more likely to have a GCS <15 compared with those who were CrAg- (Fig 2). Not shown in Fig 2, one CrAg+ subject and one CrAg- subject had a GCS in the severe (≤8) range.

### CSF CrAg positivity

**Fig 3:**
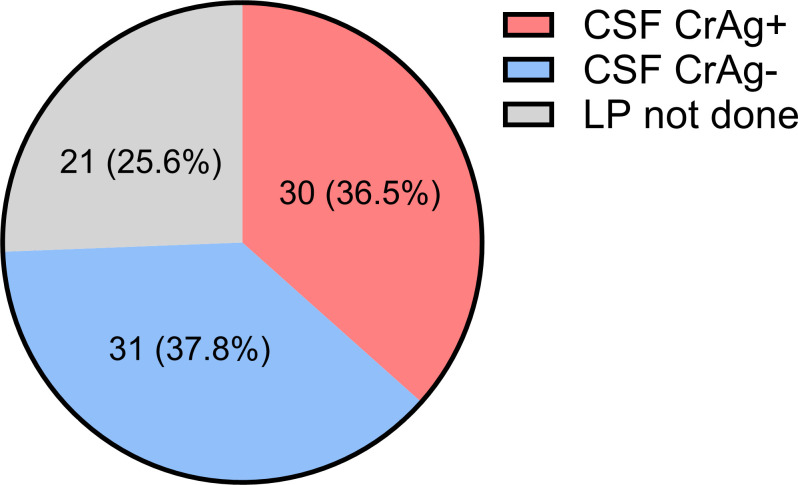
CSF CrAg in subjects with serum cryptococcal antigenemia. Subjects (n=82) who were serum CrAg+ were offered lumbar punctures (LP). For those who consented, CrAg was tested in the CSF. Numbers in the pie chart show the number of subjects and percentage of subjects in each category.

Of the 82 subjects who were CrAg+, 61 (74.3%) consented to having a lumbar puncture performed. The CSF CrAg was positive in 30/61 (49.2%) of those patients (Fig 3). Not shown in Fig 3, 15/30 (50%) of the CSF CrAg+ subjects had a GCS<15.

## Discussion

The prevalence of cryptococcosis in West Africa has not been well studied. In Freetown, Sierra Leona, 8/170 (4.7%) patients with a CD4 count <100 cells/µl were CrAg+ [14]. In a study of antiretroviral therapy naïve HIV-infected patients with a CD4 count ≤200 cells/µl in Benin City, Nigeria, 19/150 (12.7%) were CrAg+ [15]. An analysis of 47 published studies produced an estimated 6% global prevalence of serum CrAg+ among HIV-infected outpatients with a CD4 count <100 cells/µl [16]. In our study, conducted in Monrovia, Liberia, 40.7% of subjects were serum CrAg+. This high prevalence likely was biased by three factors. First, over half the study subjects were recruited from the inpatient setting. Second, nearly half of the CrAg+ subjects had an abnormal GCS. Third, study enrollment may have been biased towards sicker patients given that testing for CD4 and CrAg was not otherwise available. Without these biases, it is likely that the prevalence would have been lower.

Nearly 75% of the subjects with cryptococcal antigenemia had a lumbar puncture and of those, almost half had a positive CSF CrAg test. This high level of CSF antigen was likely biased by the same factors that contributed to the high prevalence of serum cryptococcal antigenemia noted above. Unfortunately, at the time of the study, JFK Hospital did not have the capacity to perform CSF microscopy or cultures. However, it is likely that the vast majority of those with a positive CSF CrAg test had cryptococcal meningitis. Interestingly, in subjects with cryptococcal antigenemia, having a GCS<15 was not predictive of a positive CSF CrAg test.

Fluconazole treatment of patients with CD4 counts <100 cells/µl who have asymptomatic cryptococcal antigenemia has been shown to decrease mortality [3,17]. In our study, all but one CrAg+ subject survived to day 30 of the study. We speculate that providing fluconazole to CrAg+ subjects contributed to the high survival rate, especially among the patients with cryptococcal meningitis. In a study of HIV+ persons in Uganda, all five CrAg+ persons with a CD4 count less than 100 who were not treated with fluconazole died within two months [17]. In contrast, 30 month survival was 71% in the 21 individuals promptly treated with fluconazole. Unfortunately, we do not have longer term follow-up of the subjects in our study.

To our knowledge, this study is to the first to compare cryptococcosis prevalence in individuals infected with HIV-1 versus HIV-2, both of which are endemic in West Africa [13,18]. We found HIV-2+ subjects were less likely to be CrAg+. The reasons for this finding are speculative. Compared with HIV-1 infection, HIV-2 infection progresses slower on average and has lower viral loads [18]. Whereas all of our patients had CD4^+^ T cells ≤200, it is possible that the counts were lower in the HIV-1+ population compared with those infected with HIV-2. Interestingly, although the numbers were small, the protective effect of HIV-2 on cryptococcal antigenemia extended even to those dually infected with HIV-1 and HIV-2. HIV-2 has been reported to inhibit HIV-1 gene expression and decrease HIV-1-associated pathogenesis during co-infection [19].

Our data demonstrate the utility of sequential point-of-care testing measuring CD4 counts and CrAg in settings where flow cytometric CD4 testing is not available. The LFA kits require minimal training, are simple to use, and give results in less than an hour [3,9,10]. A low CD4 count identifies patients at high risk not only for cryptococcosis but also for other opportunistic infections. Thus, the information obtained informs clinical decision making, such as the need for trimethoprim-sulfamethoxazole prophylaxis [4,11]. Unfortunately, the availability of CD4 testing in low-resource settings has decreased as funders have shifted towards strategies emphasizing viral suppression [20, 21]. The LFA tests are unavailable in many low-income areas of the world where even the modest costs of the tests make them unaffordable. Based on the results of a recent survey, it was estimated that CrAg testing is frequently accessible to only about a quarter of the African population [2]. Compounding this problem is the lack of access to amphotericin B and 5-flucytosine, drugs that have been shown to improve survival compared to fluconazole when given as initial treatment of cryptococcal meningitis [22, 23]. Clearly there is a critical need for greater diagnostic capacity and access to life-saving antifungal medications. This is certainly true in Liberia where CrAg testing, amphotericin B, and 5-flucytosine generally are not available.

The Visitech CD4 Advanced Disease LFA has been validated in field tests conducted in Malawi, Zimbabwe, and the Democratic Republic of Congo [24]. The sensitivity and specificity were 95% and 82%, respectively, compared with simultaneously obtained flow cytometry. Most misclassifications occurred with samples near the 200 CD4 cells/µl threshold. For those patients with a CD4 count less than 200 cells/µl (the group most at risk for cryptococcosis), the sensitivity increased to 98%. As we did not have access to a working flow cytometer, we were unable to perform similar studies to validate the accuracy of the CD4 LFA as part of our study.

Rare instances of false-negative CrAg test results have been reported due to the postzone phenomenon [25]. This is thought to be due to the presence of excess antigen binding the detection antibody and can be obviated by repeating the test using diluted serum. False-negative CrAg testing can also be seen early in infection when the fungal burden is low and in cases where the Cryptococcus strain sheds low amounts of capsule. False positive tests are uncommon but have been reported due to infections with fungal species taxonomically related to Cryptococcus [26]. In our study, CrAg tests were not titered and cultures were not performed. Thus, some false-positive and false-negative CrAg tests may not have been recognized. Finally, although LFAs are simple to perform, user mistakes in performing or reporting the tests could lead to erroneous data being recorded.

The use of a point of care urine lipoarabinomannan LFA to assist in the diagnosis of active tuberculosis is recommended by the WHO for HIV-positive individuals with risk factors including low CD4^+^ T cell counts [27]. In a cluster randomized trial performed in Ghana, lipoarabinomannan testing reduced the time to diagnosis of tuberculosis and initiation of antituberculosis therapy [28]. This raises the possibility of using sequential point of care testing to detect multiple opportunistic infections in persons with advanced HIV disease. In this regard, a highly sensitive and specific LFA to detect *Histoplasma capsulatum* antigen in serum is commercially available [29].

In conclusion, sequential point of care testing enables early detection of cryptococcosis and other opportunistic infections in persons with HIV/AIDS and blood CD4 T cell counts ≤200 cells/µl. In our study conducted in a large teaching hospital in Monrovia, Liberia, a strikingly high percentage of subjects with blood CD4 T cell counts ≤200 cells/µl were serum CrAg+. Moreover, nearly half of subjects with serum cryptococcal antigenemia who had a lumbar puncture were found to be CSF CrAg+. As diagnostic testing informs life-saving therapies, it is imperative that these assays are made readily available in resource-poor settings.

## Supporting information

S1 Text Client information sheet, consent form, study questionnaire and clinical data collection tool. (DOCX)

S1 Table Original data file for subjects enrolled in the study with CD4 counts of ≤200. (XLSX)
