# Supplementary material for: Use of sequential lateral flow assays to diagnose cryptococcal infection among people living with HIV in Monrovia, Liberia
Source: PLoS Negl Trop Dis. 2025 Apr 8;19(4):e0013008. doi: 10.1371/journal.pntd.0013008 (PMC12011301; doi:10.1371/journal.pntd.0013008)
Supplement: S1 Text — (DOCX) [file pntd.0013008.s001.docx]

**The prevalence of cryptococcal infection amongst HIV infected patients with CD4 count < 200 cell/ L in the medical department and IDC at J.F.K. Medical Center, Monrovia, Liberia**

**One Year Cross-sectional Prospective study**

**BY:**

**Yassah M. Barclay-Korbo^1,2^,**

**Prof. Stuart M. Levitz^3^,**

**Ibrahim Ajam ^2^,**

**Co authors**

**Prof. Sunny Chinenye ^1,2^**

**Mukhtar A. Adeiza^1,2^**

**Ian Wachekwa^1,2^,**

**Christopher Dike^1^,**

**Flinhway Hessou-Dickson^1^,**

**Nenyarker A. Vaye^1^**

**Isaac Kekulah^1^,**

**James Luke. Garlo ^1^**

**^]^**

1. **Department of Internal Medicine, John F. Kennedy Medical Center, P.O. BOX 1973, Monrovia, Liberia.**
2. **Faculty of Internal Medicine, Liberia College of Physicians and Surgeons, 12^th^Street & Russell Avenue, Monrovia, Liberia.**
3. **Professor of Medicine and Microbiology and Physiological systems University of Massachusetts Medical School 364 Plantation Street, LRB 317 Worcester, MA 01605 Stuart.Levitz@umassmed.edu**

**Client information sheet**

**We, from the Department of Internal Medicine at the John F. Kennedy Medical Center, are conducting** a study to determine the Prevalence of Cryptococcal infection amongst HIV infected patient in the medical department and Infectious disease clinic (IDC) at the JFK Medical Center. The study aims are to screen for Cryptococcal infection amongst patients attending the medical department, the IDC at JFK and other hospitals mention above.

Initial assessment will involve filling a form with the help of an interviewer concerning the demographic characteristics (age, sex, address, and educational status), date of diagnosis, duration diagnosis, and number of sexual partners before and after diagnosis among others and any positive History of HIV among children or sexual partners.

The information obtained will be used for scientific purposes. The findings will further help policy makers at the Ministry of Health to design interventions aimed at enhancing screening of cryptococcal infections among HIV positive patients attending J.F.K Medical Center, Monrovia Liberia and other hospitals.

Confidentiality will be observed, your initials as opposed to names will be used to strengthen this. Your name will not be used in reports for publications resulting from this study.

There is no potential risk. The only discomfort will be the time talking to you and screening for Cryptococcal infection (serum and Cerebral spinal fluid CRAG), HIV, Complete blood count, CD4 count, and IgM and IgG for Toxoplasma.

Participation in the study is voluntary and you are free not to take part or to opt out of the study at any point in time. Your refusal to take part will not alter your treatment in this or any other hospital. If you accept to take part, you will be required to sign or thumbprint at the bottom of this form. If you have any questions concerning the study please contact **Yassah M. Barclay-Korboi**, Department of Internal Medicine, JFK Hospital or on **Telephone No. XXXXXXX.**

**Statement of consent**

I agree to participate in a study entitled “**The prevalence of Cryptococcal infection at J.F.K Medical Center** being conducted by Yassah M. Barclay- Korboi and the Departments of Internal Medicine and Infectious disease clinic (IDC) at the JFK Medical Center.

The study has been explained to me in a manner and language that I understand. I understand that if I have any questions at a later stage ,’]I can address them to the person whose address I know. I therefore consent to the study by appending my signature.

Signature……………………………………………… (Participant)

Date…………………………………………

I have explained the purpose of the study as well as the benefits, procedure to follow and risks involved to the patient. To the best of my knowledge she/he understands what the study involves.

Signature…………………………………………………… (Investigator)

Date………………………………………….

**Study Questionnaire**

**Socio-demographic data**

Initials: __________________ Hospital No: __________________ Study No.: _____________

1. Age (Completed years Sex: Male Female
2. Place of residence: 1. Rural 2. Urban ----------------------------------------------
3. Level of education: 1. None 2. Primary level 3. Secondary level 4. Tertiary
4. Are you employed? 1. Yes 2. No if yes, what is the occupation ____________
5. Religion: 1. Muslim 2. Christian 3. None or Others-----------------------------------

**CLINICAL DATA COLLECTING TOOL**

1. **Mode of diagnosis 1).** VCCT  **2.** ANA 3.ill health 4. Tissue donation

(If HIV positive)

1. **Date of diagnosis of HIV**---------------------------------
2. **Symptoms**: Headache ___Seizure____ Neck pain_____ Fever ______Consciousness_____

**Meningeal sign__________________**

1. **Number of sexual partners (Before HIV Diagnosis):**

- 1 2. 2-3 3. 3-4 4. ≥ 5 _____________________________

1. **What medication do you take?**

1). CTX or Dapsone

2). ART:

a. TDF +3TC+EFV

b). TDF + 3TC+ NVP

cTDF+3TC+DTG

3). Fluconazole

**4) None** -------------------------

1. HIV Clinical stage ---------------------

2. Cryptococcosis --------------------------------

3. Toxoplasmosis -----------------------------

4. TB---------------------------------------------

**5. Others ___________________________________________________**

**xi. INVESTIGATIONS:**

1. **HIV test ________________ and Types_____________________**

**2) Serum CRAG_________________**

- **Lumbar Puncture CSF CRAG--------------------------**
- **Opening Pressure___________________**

**2).CD4-------------------------------**

**3). Viral load HIV____________________________**

**4) CBC: a. WBC_______________________**

**b. Lymphocytes _________________**

**c. Neutrophilia_______________**

**d. Hgb-------------------------------**

**e. PLT-----------------------**

**5) Toxoplasmosis IgM and IgG---------------------------------------------------**

**6) Adenosine Deaminase (ADA) of the CSF--------------------------------------------**

**____________________________________________________________________________________________________________________________________________________________**

**_____________________________________________________________________**

**Completed by:**

**Name: ____________________________ Signature: ______________________________**
